# Extracellular Matrix Signals as Drivers of Mitochondrial Bioenergetics and Metabolic Plasticity of Cancer Cells During Metastasis

**DOI:** 10.3389/fcell.2021.751301

**Published:** 2021-10-18

**Authors:** Félix A. Urra, Sebastián Fuentes-Retamal, Charlotte Palominos, Yarcely A. Rodríguez-Lucart, Camila López-Torres, Ramiro Araya-Maturana

**Affiliations:** ^1^Laboratorio de Plasticidad Metabólica y Bioenergética, Programa de Farmacología Molecular y Clínica, Instituto de Ciencias Biomédicas, Facultad de Medicina, Universidad de Chile, Santiago, Chile; ^2^Network for Snake Venom Research and Drug Discovery, Santiago, Chile; ^3^Instituto de Química de Recursos Naturales, Universidad de Talca, Talca, Chile

**Keywords:** OXPHOS (oxidative phosphorylation), integrin, TCA cycle, ECM stiffness, migrastatics, migrating cancer cells, metabolic shift

## Abstract

The role of metabolism in tumor growth and chemoresistance has received considerable attention, however, the contribution of mitochondrial bioenergetics in migration, invasion, and metastasis is recently being understood. Migrating cancer cells adapt their energy needs to fluctuating changes in the microenvironment, exhibiting high metabolic plasticity. This occurs due to dynamic changes in the contributions of metabolic pathways to promote localized ATP production in lamellipodia and control signaling mediated by mitochondrial reactive oxygen species. Recent evidence has shown that metabolic shifts toward a mitochondrial metabolism based on the reductive carboxylation, glutaminolysis, and phosphocreatine-creatine kinase pathways promote resistance to anoikis, migration, and invasion in cancer cells. The PGC1a-driven metabolic adaptations with increased electron transport chain activity and superoxide levels are essential for metastasis in several cancer models. Notably, these metabolic changes can be determined by the composition and density of the extracellular matrix (ECM). ECM stiffness, integrins, and small Rho GTPases promote mitochondrial fragmentation, mitochondrial localization in focal adhesion complexes, and metabolic plasticity, supporting enhanced migration and metastasis. Here, we discuss the role of ECM in regulating mitochondrial metabolism during migration and metastasis, highlighting the therapeutic potential of compounds affecting mitochondrial function and selectively block cancer cell migration.

## Introduction

Currently, it is known that the activation of oncogenes such as c-Myc, Oct, and K-Ras (Jose et al., 2011; Hu et al., 2012; Sancho et al., 2015) and cellular sensors such as mTOR, AMPK, and HIF1α participate in the metabolic adaptations that support the primary tumor growth (Massagué and Obenauf, 2016; Valcarcel-Jimenez et al., 2017; Desbats et al., 2020; Moldogazieva et al., 2020); however, how the cancer metabolism changes during metastasis remain less well known. During the initiation of metastatic cascade, cancer cells interact with the extracellular matrix (ECM) through cell surface receptors (e.g., integrins). The ECM is composed of collagens, proteoglycans, and glycoproteins (such as laminin, fibronectin, elastin, and tenascins). Tumor-associated ECM is dynamically modified by matrix metalloproteases (MMP), producing alterations of tissue stiffness, porosity, and organization (Lu et al., 2012), being biochemically and mechanically different to normal ECM (Pickup et al., 2014). These abnormal changes in ECM promote cellular transformation and metastasis, facilitate tumor associated angiogenesis and inflammation, and determine the chemotherapy efficacy (Lu et al., 2012; Northcott et al., 2018; Deville and Cordes, 2019).

**FIGURE 1:**
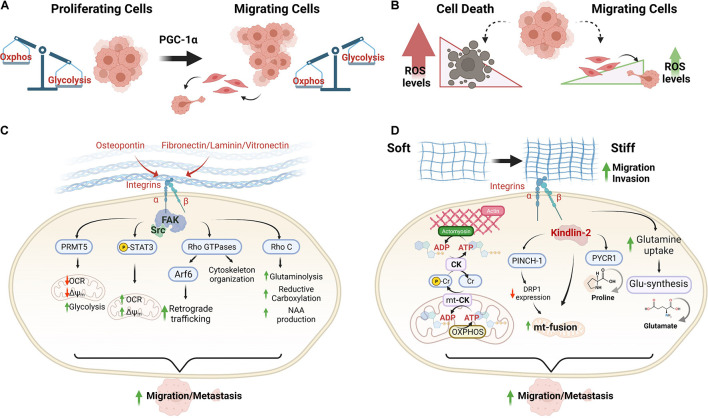
Extracellular matrix signals induce metabolic plasticity for increasing the mitochondrial bioenergetics of cancer cells during the metastastic cascade. **(A,B)** Differential metabolic phenotypes between proliferating and migrating cancer cells. PGC-1α has been recognized as an essential regulator of the metabolic shifts that support metastasis. **(C)** Mitochondrial adaptations driven by ECM components and mediated by integrin and FAK signaling in cancer cells. **(D)** Adaptations of mitochondrial bioenergetics driven by ECM stiffness in migrating cancer cells. Abbreviations: OXPHOS, oxidative phosphorylation; OCR, oxygen consumption rate; Δψm, mitochondrial membrane potential; pCr, phosphocreatine; CK, creatine kinase; mt-CK, mitochondrial creatine kinase; and NAA, N-acetylaspartate.

For initiating migration, cancer cells depend on their metabolic plasticity for adapting the energy production according to changes in ECM (Lipinski et al., 2016), in which mitochondria take over a crucial role for supporting metastasis formation (Scheid et al., 2021; Zanotelli et al., 2021; Figures 1A,B). In this review, we discuss the role of ECM components and ECM mechanical changes in regulating metabolic plasticity and mitochondrial bioenergetics in migration and metastasis.

## Metabolic Plasticity During Metastasis: Role of Mitochondrial Bioenergetics

Upon metabolic stress, energy demands are supplied through dynamic changes in the metabolism. This process, known as metabolic plasticity, allows cancer cells to remodel the energy-producing pathways [e.g., metabolic shifts between glycolysis versus oxidative phosphorylation (OXPHOS)], preference of mitochondrial oxidable substrates (e.g., pyruvate, glutamine versus fatty acid), and synthesis of intermediates of the tricarboxylic acid (TCA) cycle (e.g., induction of reductive carboxylation versus oxidative decarboxylation), which depend on changes of substrate availability, such as oxygen, glucose, and amino acids (Urra et al., 2016b). To metastasize, cancer cells must perform metabolic adaptations to detachment from ECM, local migration and invasion, intra- and extra-vasation in blood, local invasion to into secondary sites, and formation of a secondary tumor (Celià-Terrassa and Kang, 2016; Scheid et al., 2021).

The resistance to detachment-induced cell death (anoikis) and overcoming the growth signals received through their attachment to ECM are important markers for the initial steps of metastasis (Simpson et al., 2008). Under anoikis, a metabolic remodeling toward increased pyruvate utilization promotes the migration of highly invasive ovarian cancer cells (Caneba et al., 2012). In line with this, under metabolic stress, AMPK promotes the PDH activity, which catalyzes pyruvate to acetyl-CoA, maintaining the influx of substrates for TCA cycle functioning, supporting a metastatic phenotype (Cai et al., 2020). This correlates with high glutamine consumption for mitochondrial ATP synthesis (Yang et al., 2014; Fiorillo et al., 2021). Consequently, the inhibition of mitochondrial function reduces the invasive capacity of these cancer cells (Kim and Wirtz, 2011; Caneba et al., 2012; Yang et al., 2014). Besides, cancer cells manage redox homeostasis and growth using reductive carboxylation dependent on glutamine-derived α-ketoglutarate (α-KG), while adapting to an anchorage-independent phenotype (Jiang et al., 2016).

After escape from the primary tumor mass and intravasation, circulating cancer cells rewire their metabolism to survive, controlling the mitochondrial reactive oxygen species (mtROS) scavenging (Elia et al., 2018). Recently, differential utilization of glycolysis and OXPHOS between proliferating and migratory/invasive cancer cells has highlighted the participation of mitochondria during metastasis. Invasive cancer cells use the transcription coactivator peroxisome proliferator-activated receptor gamma, coactivator 1 alpha (PPARGC1A, also known as PGC-1α) to enhance mitochondrial biogenesis and OXPHOS, being an essential event for functional motility and metastasis in breast cancer cells (LeBleu et al., 2014). In addition, subpopulations of cancer cells able to generate metastasis require a high production of mitochondrial superoxide (Porporato et al., 2014), which is obtained by an exaggerated TCA cycling (Porporato and Sonveaux, 2014; Porporato et al., 2014). The PGC1a-driven metabolic adaptations with increased OXPHOS are essential to tumorigenesis, showing a positive influence on metastasis in several cancers, such as breast (Cai et al., 2016; Luo et al., 2016b; Andrzejewski et al., 2017; Pacheco-Velázquez et al., 2018), hepatocellular (Li et al., 2016), colorectal (Yun et al., 2019), endometrial (Chen et al., 2020), prostate (Tennakoon et al., 2014), pancreatic cancers (Sancho et al., 2015), and in some models of melanoma (Vazquez et al., 2013). Despite the above, the PGC-1α overexpression decreases cellular invasiveness in prostate and melanoma (Luo et al., 2016a; Torrano et al., 2016), showing that the link between increased PGC-1α expression, mtROS, and metastasis still remains controversial and suggests specific roles in tumorigenesis dependent on cancer type (LaGory et al., 2015; Piskounova et al., 2015; Liu et al., 2017).

Mitochondrial ATP and ROS are essentials for supporting metastatic signaling (Lu et al., 2012; LeBleu et al., 2014; Porporato et al., 2014; Ryu et al., 2020). A controlled mtROS increase triggers an invasive behavior by stimulating Src signaling, which in turn induces pyk2 expression, a tyrosine kinase of the FAK family involved in cytoskeletal remodeling and migration (Du et al., 2001). In the same line, Src increases the metabolic status of metastatic cells by phosphorylation of residues of respiratory complexes, enhancing the activities of the electron transport chain (ETC) and the PFKFB3 complex, leading to an increase of the fluxes of glycolysis, non-oxidative pentose phosphate pathway and TCA cycle (Ma et al., 2020).

Recent reports highlight the key role of α-KG in the metabolic shifts that promote metastasis (Elia et al., 2018). α-KG is synthesized from pyruvate oxidation or glutaminolysis and metabolized by α-ketoglutarate dehydrogenase (Armstrong et al., 2014), which is essential for cancer proliferation and survival under hypoxia and OXPHOS dysfunction (Burr et al., 2016; Vatrinet et al., 2017; Cardenas et al., 2020). Increased α-KG levels trigger a strong epigenetic reprogramming that enhances the adaptation of cancer cells to a hostile environment through the activation of histone and/or DNA demethylases (Rinaldi et al., 2018). Beyond the essential role of glutaminolysis in proliferation by supporting the nucleotide synthesis (Metallo et al., 2012; Mullen et al., 2012), glutamine regulates the MMP expression dependent on the oncogenic transcription factor ETS1, which triggers an invasive phenotype by a not fully elucidated mechanism (Prasad and Roy, 2021). Finally, cancer cells that reach a distant organ, they colonize the new environment, establishing new cell-matrix interaction, ECM remodeling, and micrometastasis formation in a bioenergetics-dependent manner (Elia et al., 2018; Schild et al., 2018; Scheid et al., 2021). In particular, breast cancer cells colonizing lungs utilize the proline cycle to obtain FADH_2_, which can be oxidized by ETC to produce mitochondrial ATP (Elia et al., 2017). In addition, the metastasizing breast and oral squamous cancer cells have high bioenergetics plasticity to support ATP synthesis by glycolysis and fatty acid-dependent OXPHOS, suggesting an selective rewiring of energy substrate preference (Andrzejewski et al., 2017; Pascual et al., 2017). Therefore, different mitochondria-dependent metabolic adaptations occur during migration, invasion, and colonization; however, they all converge to supply mitochondrial ATP production, revealing an essential role of bioenergetics in metastasis.

## Extracellular Matrix Components That Modulate the Mitochondrial Bioenergetics in Metastasis

The migration of cancer cells through the extracellular matrices requires cell-ECM interactions mediated by non-collagenous ECM glycoproteins fibronectin and laminin (Parsons et al., 2010). These two ECM glycoproteins bind specific collagens and proteoglycans, interacting with integrin receptors in the plasma membrane of cancer cells. Integrins are heterodimers of type 1 membrane-spanning glycoproteins composed of one α and one β subunit, interacting with the ECM to produce a dynamic link between the extracellular adhesion molecules and the intracellular actin cytoskeleton, thereby promoting intracellular signaling cascades (Blandin et al., 2015). The aggregation of ECM proteins, integrins, cytoskeletal proteins, and signaling kinases form structures known as focal adhesion complexes (Parsons et al., 2010). The integrin ligation and clustering activate focal adhesion kinase (FAK), which in-turn activates Src-family kinases and this increases downstream pathways for promoting survival and motility, contributing to metastasis (Cance et al., 2000; Lark et al., 2005; Mitra and Schlaepfer, 2006).

Activation of integrin signaling controls the metabolism, promoting metabolic shifts that support migration and metastasis (Figure 1C). Osteopontin is a small N-linked glycoprotein that binds αvβ3 integrin. This interaction reduces the mitochondrial membrane potential (Δψm) and oxygen consumption rate (OCR), producing a metabolic shift toward glycolysis that supports migration and growth in glioblastoma cells (Che et al., 2021). This integrin-dependent metabolic shift is mediated by FAK/protein arginine methyltransferase 5 activation (Che et al., 2021). Notably, contrary effects of other integrin ligands such as vitronectin, laminin, and fibronectin have been described for stimulating the mitochondrial function, increasing the maximum reserve and respiratory capacity in endothelial cells by STAT3 activation. Integrin ligands induce STAT3 translocation to mitochondria for stimulating OXPHOS function (Visavadiya et al., 2016) and recently, was described that ECM-integrin-FAK-STAT3 signaling promotes migration in cancer cells (Yang et al., 2021). This indicates a possible differential metabolic regulation by ECM in migrating cells.

The Rho family of small GTPases integrates ECM-integrin signals for controlling cell cycle progression, migration, and actin cytoskeleton dynamics, which are relevant during metastasis (Phuyal and Farhan, 2019). Notably, some small GTPases participate in the mitochondrial fission during intrinsic apoptosis and mitophagy (Hammerling et al., 2017), and glutamine metabolism (Dorai et al., 2016). RhoC promotes glutamine uptake for maintaining α-KG-dependent reductive carboxylation in SUM149 cells, an inflammatory breast cancer model. SUM149 cells exhibit metabolic abnormalities such as high aerobic glycolysis, low mitochondrial respiratory capacity, and a large reductive carboxylation flux from glutamine-derived α-KG to citrate under normal culture conditions, which are supported by RhoC (Wynn et al., 2016). *N*-acetylaspartate (NAA), a storage metabolite for acetate, is synthesized from aspartate and acetyl-CoA by aspartate-*N*-acetyltransferase (Asp-NAT) or via hydrolysis of *N*-acetyl-aspartylglutamate. RhoC promotes the NAA production in SUM149 cells by regulation of Asp-NAT levels. Since the changes in the tumoral microenvironment may determine nutrient shortage, NAA storage may help promote survival and to accommodate varying nutritional needs during the diverse steps of the metastatic process (Wynn et al., 2016).

Regulatory mechanisms of mitochondrial distribution mediated by β1-integrin have been described for highly invasive cancer cells. Upon integrin recycling, the small GTPase Arf6 promotes an AMAP1–ILK signaling pathway essential for the formation of mature focal adhesions in invasive cancer cells. This blocks the RhoT1-TRAK2 association, reducing the mitochondrial retrograde trafficking without changes in the mitochondrial mass and OCR, and favoring collagen I-stimulated cell invasion (Onodera et al., 2018). In migrating ovarian cancer cells, lamellipodia have increased local mitochondrial mass, elevated OCR, and relative ATP concentration. Notably, this is dependent on an increased pseudopodial AMPK activity that maintains the cytoskeletal dynamics, migration, and the invasion of three-dimensional ECM (Cunniff et al., 2016). Consistent with this, mitochondrial Rho GTPase (Miro1) involved in the mitochondrial trafficking, also controls the ATP/ADP ratio at the cortex, promoting lamellipodia protrusion and membrane ruffling in migrating cells (Schuler et al., 2017). Collectively, this evidence suggests that local mitochondrial accumulation in the leading edge lamellipodia has bioenergetics implications in migrating cancer cells by supporting membrane protrusion and focal adhesion stability.

## Mechanosignalings From Extracellular Matrix That Modulate the Mitochondrial Bioenergetics and Metabolism Plasticity During Metastasis

During metastatic dissemination, cancer cells adapt to structurally and mechanically different ECM in the primary tumor. The ECM remodeling in a tumor is characterized by increased ECM deposition, fiber alignment, and crosslinking, modifying the stiff tumor microenvironment. This promotes active cancer progression and metastasis increased by integrin signaling (Egeblad et al., 2010; Winkler et al., 2020). Unlike normal tissue, several solid cancers exhibit a more ECM stiffness (Kawano et al., 2015) and have dense and align collagen fibers, which favor the exit of migrating cancer cells from the primary tumor (Provenzano et al., 2006, 2008).

During microenvironment transitions with variations on mechanical cues, migrating cancer cells regulate the metabolism for supplying the energetic needs (Papalazarou et al., 2020; Zanotelli et al., 2021; Figure 1D). Stiff ECM promotes mitochondrial fusion by activation of β1-integrin/kindlin-2 (an integrin-binding protein) signaling (Chen et al., 2021). In this condition, concomitantly occurs the activation of a signaling by β1-integrin/PINCH-1, a focal adhesion protein whose level is increased in response to ECM stiffening, decreasing DRP1 GTPase expression and mitochondrial fission (Chen et al., 2021). Conversely, soft ECM induces up-regulation of DRP1 expression and mitochondrial fission, reducing the spreading of cancer cells (Chen et al., 2021). Although DRP1 knockdown prevents the ECM softening-induced mitochondrial fission, it lacks effects on spreading, suggesting that other molecular components may participate in this signaling. Moreover, details on the impact of mitochondrial bioenergetics during migration mediated by β1-integrin/PINCH-1 or β1-integrin/kindlin-2 remain unknown. A possible link of this signaling to mitochondrial metabolism may be the reprogramming of proline metabolism, which is critical for tumor growth. PINCH-1, highly expressed in lung adenocarcinoma, promotes proline synthesis through the regulation of mitochondrial dynamics. Knockout of PINCH-1 increases DRP1 expression and mitochondrial fragmentation, which suppresses kindlin-2 mitochondrial translocation, and interaction with pyrroline-5-carboxylate reductase 1, resulting in inhibition of proline synthesis and cancer cell proliferation (Guo et al., 2019, 2020).

Mechanical regulation of cytoskeletal remodeling during spreading and migration involves a metabolic shift toward increased OXPHOS, which is necessary for membrane ruffling in breast cancer cells (Wu et al., 2021). Under ECM stiffness, the high energy demand is supplied by the phosphocreatine (pCr)–creatine kinase (CK) system in pancreatic ductal adenocarcinoma (PDAC) cells. PDAC is associated with extensive matricellular fibrosis and more aggressiveness (Bailey et al., 2016; Tian et al., 2019; Papalazarou et al., 2020). In these cells, K-RAS activating mutations drive high metabolic plasticity, conferring adaptive metabolic mechanisms for consuming alternative energy sources (Kerr et al., 2016). A metabolic remodeling induced by a stiffer matrix has been described for PDAC cells which supports migration and metastasis. In matrix stiffness, PDAC cells shunt L-arginine toward the creatine biosynthesis pathway, increasing the ATP turnover and pCr reaction by CK (Papalazarou et al., 2020). The high pCr-CK activity promotes elongated mitochondria, increasing mitochondrial mass and Δψm to support ATP production by OXPHOS (Papalazarou et al., 2020). Remarkably, this mitochondrial subpopulation enriches the pseudopods of PDAC cells invading the ECM.

In solid tumors, collaborative metabolic shifts between stroma and epithelial cell populations maintain a continuous supply of energetic substrates (Martinez and Smith, 2021). Cancer-associated fibroblasts (CAFs) secrete lactate, which increases mitochondrial mass and activity by SIRT1-dependent PGC-1α activation in cancer cells and promotes mitochondrial transfer from CAF (Ippolito et al., 2019). Moreover, increased ECM stiffness stimulates the expression of stromal glucose transporter Glut1 and monocarboxylate transporters MCT4, increasing lactate production and glucose uptake by mammary fibroblasts (Ponce et al., 2021). In this condition, mammary stromal cells generate soluble factors that stimulate epithelial breast migration in a stiffness-dependent manner (Ponce et al., 2021). Moreover, tumor niche stiffening induces a differential switch in amino acid metabolism involving a change in carbon fluxes in cancer and stromal cells (Bertero et al., 2019). In CAFs, ECM stiffness promotes aspartate synthesis from glutamine-derived carbon and glutamate synthesis from glutamine-derived carbon in cancer cells, failing to fill the TCA cycle and aspartate synthesis (Bertero et al., 2019). Differential roles of these amino acids in CAF and cancer cells have been described. Aspartate promotes cancer cell proliferation by participating in the nucleotide biosynthesis pathway, while glutamate feeds the glutathione synthesis for controlling intratumorally redox homeostasis. Notably, co-targeting of glutaminase and the aspartate/glutamate transporter SLC1A3 in tumors blocks cancer progression and metastasis *in vivo* (Bertero et al., 2019). Therefore, this evidence suggests new anticancer strategies that can overcome the ECM mechanosignalings-driven metabolic adaptations in solid tumors.

## Migrastatic Agents That Promote Mitochondrial-Extracellular Matrix Disruption in Cancer Cells

Although the metastasis is the main cause of death in patients (Riggi et al., 2018), the current chemotherapy regimens only target the tumor growth, lacking the inhibitory effects on the ability of cancer cells to invade and execute metastasis (Gandalovičová et al., 2017; Riggi et al., 2018). This highlights the need to search for novel anti-metastatic pharmacological approaches (Gandalovičová et al., 2017; Riggi et al., 2018). Migrastatic drugs have been defined as selective inhibitors of metastatic abilities with non-cytotoxic effect (Gandalovičová et al., 2017). Although some migrastatic actions of cytotoxic compounds are reported, those effects can be attributed to the induced cell death, because the direct link between migration and mitochondrial dysfunction has been not established (Song et al., 2017; Yan et al., 2017; Dong et al., 2018; Yang et al., 2018; Cheng et al., 2019; Luo et al., 2019; Gupta et al., 2021; Liu et al., 2021). Table 1 shows recent compounds reported with migrastatic effects by induction of mitochondrial dysfunction at non-cytotoxic concentrations.

**TABLE 1:** Mitochondria-affecting compounds with migrastatic effect.

Compound	Chemistry type	Mechanism of action	References
FR58P1a	Hydroquinone derivative	OXPHOS uncoupling through a protonophoric mechanism. Mitochondrial fragmentation and dysfunction, promote AMPK activation in a SIRT1-dependent manner, leading to selective inhibition of fibronectin-dependent adhesion and migration by decreasing β1-integrin at the cell surface in TNBC.	Urra et al., 2018
Silibinin	Polyphenolic flavonoid obtained from *Silybum marianum*	Mitochondrial fragmentation via decreased DRP1 and increased OPA1 and mitofusin 1/2 expression. Reduction of oxidized mtDNA, inhibiting the inflammasome activation, and caspase-1, and IL-1β levels. Reduced migration and invasion of the MDA-MB-231 cell line by downregulation of EMT markers (N-cadherin and vimentin) and MMP2/9 and paxillin expression.	Si et al., 2020
IR-783	Heptamethine cyanine dye	Mitochondrial fission and ATP decrease, decreasing polymerized filamentous actin and decreasing the migration in breast cancer cells	Li et al., 2019
Pictobin	A thrombin-like enzyme from *Bothrops pictus* venom	Mitochondrial fragmentation and dysfunction by increasing the mitochondrial NADH oxidation and decreasing Δψ_*m*_ and ATP levels. Reduction of fibronectin-dependent migration in lung and breast cancer cells	Vivas-Ruiz et al., 2020

Many phenolic compounds affect the mitochondrial bioenergetics, by inhibiting ETC and/or by OXPHOS uncoupling (Urra et al., 2013, 2016a, 2021; Donoso-Bustamante et al., 2020; Fuentes-Retamal et al., 2020), with different consequences on viability and proliferation of cancer cells (Urra et al., 2016b, 2017). Factors such as the degree of inhibition of ETC activity, bioenergetic profile, and metabolic plasticity of different cancer types, or subpopulations of cells in a particular cancer type, determine the anti-cancer actions (McGuirk et al., 2013; Lehuédé et al., 2016; Urra et al., 2016b). A hydroquinone derivative, named FR58P1a (Table 1), selectively uncouples OXPHOS, through a protonophoric mechanism, without exhibiting the known off-target effect on the plasma membrane potential of canonical protonophores such as FCCP and CCCP (Juthberg and Brismar, 1997; Buckler and Vaughan-Jones, 1998; Park et al., 2002). The FR58P1a-induced mitochondrial dysfunction activates the SIRT1/AMPK axis, leading to selective inhibition of fibronectin-dependent adhesion and migration by decreasing β1-integrin at the cell surface in triple-negative breast cancer (TNBC) cells (Urra et al., 2018). The prolonged migrastatic effect of FR58P1a triggers a metabolic shift toward glycolysis and mitophagy (Urra et al., 2018). The polyphenolic flavonoid silibinin promotes mitochondrial fission and impairs mitochondrial biogenesis, reducing migration and invasion of TNBC cells by reduction of epithelial to mesenchymal transition (EMT) markers (Hamarsheh and Zeiser, 2020). Since inflammation promotes tumor metastasis and can be triggered by activating the NLRP3 inflammasome via ROS-dependent mitochondrial damage (Hamarsheh and Zeiser, 2020), the silibinin-induced mitochondrial fission inhibits NLRP3 inflammasome activation and migration possibly by an antioxidant mechanism (Hamarsheh and Zeiser, 2020). Instead, IR-783 induces mitochondrial fission and a subsequent ATP drop, thereby decreasing polymerized filamentous actin, a fundamental component of filopodia at the cell surface (Hamarsheh and Zeiser, 2020).

Interestingly, several toxins isolated from snake venom exhibit selective migrastatic effects in cancer cells by interaction with integrin receptors, alterations in the actin/cytoskeleton network, and EMT inhibition (Urra and Araya-Maturana, 2017, 2020). The recently identified snake toxin pictobin induces mitochondrial fragmentation and dysfunction (Table 1), inhibiting the migration in cancer cells at non-cytotoxic concentrations (Vivas-Ruiz et al., 2020). In analogy to the effects of human thrombin on mitochondrial metabolism in platelets (Ravi et al., 2015), pictobin-induced mitochondrial dysfunction may be triggered by intracellular signaling initiated in the plasma membrane by cleavage of some receptor in cancer cells (Vivas-Ruiz et al., 2020). Taking these selected examples, mitochondrial fragmentation, and bioenergetics inhibition may represent an attractive mechanism for new anti-metastatic approaches that interfere with the extracellular cues-metabolism communication.

## Future Perspective and Conclusion

Although the determinants that lead to metabolic adaptation during dissemination and metastasis are not fully elucidated, there are common factors that link a phenotype dominated by OXPHOS, glutamine consumption and increased mtROS production in cancer cells to greater migratory and invasive potential (Porporato et al., 2014; Yang et al., 2014; Valcarcel-Jimenez et al., 2017; Davis et al., 2020). In migrating cancer cells, ECM composition and stiffness are drivers for metabolic shifts toward enhanced mitochondrial bioenergetics and local mitochondrial accumulation in the leading edge lamellipodia. This showcases mitochondria as an attractive pharmaceutical target putatively preventing cancer metastasis. Since ECM stiffness produces collapse of blood vessels in tumors (Padera et al., 2004; Primeau et al., 2005) and it impairs the abilities to deliver drugs to cancer cells (Gade et al., 2009), new drug candidates as migrastatics will require to consider these factors for obtaining *in vivo* efficacy.

## Author Contributions

FU designed and outlined the structure and contents of the review. FU, SF-R, CP, CL-T, YR-L, and RA-M contributed to the literature review, discussion, and writing of the manuscript. All authors contributed equally to the draft revisions and final approval of the version to be published.

## Conflict of Interest

The authors declare that the research was conducted in the absence of any commercial or financial relationships that could be construed as a potential conflict of interest.

## Publisher’s Note

All claims expressed in this article are solely those of the authors and do not necessarily represent those of their affiliated organizations, or those of the publisher, the editors and the reviewers. Any product that may be evaluated in this article, or claim that may be made by its manufacturer, is not guaranteed or endorsed by the publisher.

## References

[B1] Andrzejewski S. Klimcakova E. Johnson R. Tabariès S. Annis M. McGuirk S. (2017). PGC-1α promotes breast cancer metastasis and confers bioenergetic flexibility against metabolic drugs. *Cell Metab.* 26 778–787. 10.1016/j.cmet.2017.09.006 28988825

[B2] Armstrong C. T. Anderson J. L. Denton R. M. (2014). Studies on the regulation of the human E1 subunit of the 2-oxoglutarate dehydrogenase complex, including the identification of a novel calcium-binding site. *Biochem. J.* 459 369–381. 10.1042/bj20131664 24495017

[B3] Bailey P. Chang D. K. Nones K. Johns A. L. Patch A.-M. Gingras M.-C. (2016). Genomic analyses identify molecular subtypes of pancreatic cancer. *Nature* 531 47–52. 10.1038/nature16965 26909576

[B4] Bertero T. Oldham W. M. Grasset E. M. Bourget I. Boulter E. Pisano S. (2019). Tumor-stroma mechanics coordinate amino acid availability to sustain tumor growth and malignancy. *Cell Metab.* 29 124–140. 10.1016/j.cmet.2018.09.012 30293773 PMC6432652

[B5] Blandin A. Renner G. Lehmann M. Lelong-Rebel I. Martin S. Dontenwill M. (2015). β1 integrins as therapeutic targets to disrupt hallmarks of cancer. *Front. Pharmacol.* 6:279. 10.3389/fphar.2015.00279 26635609 PMC4656837

[B6] Buckler K. J. Vaughan-Jones R. D. (1998). Effects of mitochondrial uncouplers on intracellular calcium, pH and membrane potential in rat carotid body type I cells. *J. Physiol.* 513(Pt 3) 819–833. 10.1111/j.1469-7793.1998.819ba.x 9824720 PMC2231310

[B7] Burr S. P. Costa A. S. Grice G. L. Timms R. T. Lobb I. T. Freisinger P. (2016). Mitochondrial protein lipoylation and the 2-oxoglutarate dehydrogenase complex controls hif1α stability in aerobic conditions. *Cell Metab.* 24 740–752. 10.1016/j.cmet.2016.09.015 27923773 PMC5106373

[B8] Cai F. F. Xu C. Pan X. Cai L. Lin X. Y. Chen S. (2016). Prognostic value of plasma levels of HIF-1a and PGC-1a in breast cancer. *Oncotarget* 7 77793–77806. 10.18632/oncotarget.12796 27780920 PMC5363621

[B9] Cai Z. Li C. F. Han F. Liu C. Zhang A. Hsu C. C. (2020). Phosphorylation of PDHA by AMPK Drives TCA Cycle to promote cancer metastasis. *Mol. Cell* 80 263–278. 10.1016/j.molcel.2020.09.018 33022274 PMC7534735

[B10] Cance W. Harris J. Iacocca M. Roche E. Yang X. Chang J. (2000). Immunohistochemical analyses of focal adhesion kinase expression in benign and malignant human breast and colon tissues: correlation with preinvasive and invasive phenotypes. *Clin. Cancer Res.* 6 2417–2423. 10873094

[B11] Caneba C. Bellance N. Yang L. Pabst L. Nagrath D. (2012). Pyruvate uptake is increased in highly invasive ovarian cancer cells under anoikis conditions for anaplerosis, mitochondrial function, and migration. *Am. J. Physiol. Endocrinol. Metab.* 303 E1036–E1052. 10.1152/ajpendo.00151.2012 22895781

[B12] Cardenas C. Lovy A. Silva-Pavez E. Urra F. Mizzoni C. Ahumada-Castro U. (2020). Cancer cells with defective oxidative phosphorylation require endoplasmic reticulum-to-mitochondria Ca(2+) transfer for survival. *Sci. Signal.* 13:eaay1212. 10.1126/scisignal.aay1212 32665411 PMC9387586

[B13] Celià-Terrassa T. Kang Y. (2016). Distinctive properties of metastasis-initiating cells. *Genes Dev.* 30 892–908. 10.1101/gad.277681.116 27083997 PMC4840296

[B14] Che P. Yu L. Friedman G. K. Wang M. Ke X. Wang H. (2021). Integrin αvβ3 engagement regulates glucose metabolism and migration through focal adhesion kinase (FAK) and protein arginine methyltransferase 5 (PRMT5) in glioblastoma cells. *Cancers* 13:1111. 10.3390/cancers13051111 33807786 PMC7961489

[B15] Chen K. Wang Y. Deng X. Guo L. Wu C. (2021). Extracellular matrix stiffness regulates mitochondrial dynamics through PINCH-1- and kindlin-2-mediated signalling. *Curr. Res. Cell Biol.* 2:100008. 10.1016/j.crcbio.2021.100008

[B16] Chen L. Mao X. Huang M. Lei H. Xue L. Sun P. (2020). PGC-1α and ERRα in patients with endometrial cancer: a translational study for predicting myometrial invasion. *Aging* 12 16963–16980. 10.18632/aging.103611 32920551 PMC7521515

[B17] Cheng G. Zhang Q. Pan J. Lee Y. Ouari O. Hardy M. (2019). Targeting lonidamine to mitochondria mitigates lung tumorigenesis and brain metastasis. *Nat. Commun.* 10:2205. 10.1038/s41467-019-10042-1 31101821 PMC6525201

[B18] Cunniff B. McKenzie A. Heintz N. Howe A. (2016). AMPK activity regulates trafficking of mitochondria to the leading edge during cell migration and matrix invasion. *Mol. Biol. Cell* 27 2662–2674. 10.1091/mbc.E16-05-0286 27385336 PMC5007087

[B19] Davis R. T. Blake K. Ma D. Gabra M. B. I. Hernandez G. A. Phung A. T. (2020). Transcriptional diversity and bioenergetic shift in human breast cancer metastasis revealed by single-cell RNA sequencing. *Nat. Cell Biol.* 22 310–320. 10.1038/s41556-020-0477-0 32144411

[B20] Desbats M. A. Giacomini I. Prayer-Galetti T. Montopoli M. (2020). Metabolic plasticity in chemotherapy resistance. *Front. Oncol.* 10:281. 10.3389/fonc.2020.00281 32211323 PMC7068907

[B21] Deville S. S. Cordes N. (2019). The extracellular, cellular, and nuclear stiffness, a trinity in the cancer resistome—a review. *Front. Oncol.* 9:1376. 10.3389/fonc.2019.01376 31867279 PMC6908495

[B22] Dong L. Xu W. W. Li H. Bi K. H. (2018). In vitro and in vivo anticancer effects of marmesin in U937 human leukemia cells are mediated via mitochondrial-mediated apoptosis, cell cycle arrest, and inhibition of cancer cell migration. *Oncol. Rep.* 39 597–602. 10.3892/or.2017.6147 29251335

[B23] Donoso-Bustamante V. Borrego E. A. Schiaffino-Bustamante Y. Gutiérrez D. A. Millas-Vargas J. P. Fuentes-Retamal S. (2020). An acylhydroquinone derivative produces OXPHOS uncoupling and sensitization to BH3 mimetic ABT-199 (Venetoclax) in human promyelocytic leukemia cells. *Bioorg. Chem.* 100:103935. 10.1016/j.bioorg.2020.103935 32454391

[B24] Dorai T. Pinto J. T. Cooper A. J. L. (2016). Sweetening of glutamine metabolism in cancer cells by Rho GTPases through convergence of multiple oncogenic signaling pathways. *Transl. Cancer Res.* 5 S349–S356. 10.21037/tcr.2016.07.43

[B25] Du Q. S. Ren X. R. Xie Y. Wang Q. Mei L. Xiong W. C. (2001). Inhibition of PYK2-induced actin cytoskeleton reorganization, PYK2 autophosphorylation and focal adhesion targeting by FAK. *J. Cell Sci.* 114(Pt 16) 2977–2987. 11686301 10.1242/jcs.114.16.2977

[B26] Egeblad M. Rasch M. G. Weaver V. M. (2010). Dynamic interplay between the collagen scaffold and tumor evolution. *Curr. Opin. Cell Biol.* 22 697–706. 10.1016/j.ceb.2010.08.015 20822891 PMC2948601

[B27] Elia I. Broekaert D. Christen S. Boon R. Radaelli E. Orth M. (2017). Proline metabolism supports metástasis formation and could beinhibited to selectively target metastasizing cáncer cells. *Nat. Commun.* 8:15267. 10.1038/ncomms15267 28492237 PMC5437289

[B28] Elia I. Doglioni G. Fendt S. (2018). Metabolic hallmarks of metastasis formation. *Trends Cell Biol.* 28 673–684. 10.1016/j.tcb.2018.04.002 29747903

[B29] Fiorillo M. Scatena C. Naccarato A. G. Sotgia F. Lisanti M. P. (2021). Bedaquiline, an FDA-approved drug, inhibits mitochondrial ATP production and metastasis in vivo, by targeting the gamma subunit (ATP5F1C) of the ATP synthase. *Cell Death Differ.* 28 2797–2817. 10.1038/s41418-021-00788-x 33986463 PMC8408289

[B30] Fuentes-Retamal S. Sandoval-Acuna C. Peredo-Silva L. Guzman-Rivera D. Pavani M. Torrealba N. (2020). Complex mitochondrial dysfunction induced by TPP(+)-gentisic acid and mitochondrial translation inhibition by doxycycline evokes synergistic lethality in breast cancer cells. *Cells* 9:407. 10.3390/cells9020407 PMC7072465 32053908

[B31] Gade T. P. Buchanan I. M. Motley M. W. Mazaheri Y. Spees W. M. Koutcher J. A. (2009). Imaging intratumoral convection: pressure-dependent enhancement in chemotherapeutic delivery to solid tumors. *Clin. Cancer Res.* 15 247–255. 10.1158/1078-0432.Ccr-08-0611 19118052 PMC4217124

[B32] Gandalovičová A. Rosel D. Fernandes M. Veselý P. Heneberg P. Čermák V. (2017). Migrastatics-anti-metastatic and anti-invasion drugs: promises and challenges. *Trends Cancer* 3 391–406. 10.1016/j.trecan.2017.04.008 28670628 PMC5482322

[B33] Guo L. Cui C. Wang J. Yuan J. Yang Q. Zhang P. (2020). PINCH-1 regulates mitochondrial dynamics to promote proline synthesis and tumor growth. *Nat. Commun.* 11:4913. 10.1038/s41467-020-18753-6 33004813 PMC7529891

[B34] Guo L. Cui C. Zhang K. Wang J. Wang Y. Lu Y. (2019). Kindlin-2 links mechano-environment to proline synthesis and tumor growth. *Nat. Commun.* 10:845. 10.1038/s41467-019-08772-3 30783087 PMC6381112

[B35] Gupta N. Gaikwad S. Kaushik I. Wright S. E. Markiewski M. M. Srivastava S. K. (2021). Atovaquone suppresses triple-negative breast tumor growth by reducing immune-suppressive cells. *Int. J. Mol. Sci.* 22:5150. 10.3390/ijms22105150 34068008 PMC8152242

[B36] Hamarsheh S. A. Zeiser R. (2020). NLRP3 inflammasome activation in cancer: a double-edged sword. *Front. Immunol.* 11:1444. 10.3389/fimmu.2020.01444 32733479 PMC7360837

[B37] Hammerling B. C. Najor R. H. Cortez M. Q. Shires S. E. Leon L. J. Gonzalez E. R. (2017). A Rab5 endosomal pathway mediates Parkin-dependent mitochondrial clearance. *Nat. Commun.* 8:14050. 10.1038/ncomms14050 28134239 PMC5290275

[B38] Hu Y. Lu W. Chen G. Wang P. Chen Z. Zhou Y. (2012). K-ras(G12V) transformation leads to mitochondrial dysfunction and a metabolic switch from oxidative phosphorylation to glycolysis. *Cell Res.* 22 399–412. 10.1038/cr.2011.145 21876558 PMC3257361

[B39] Ippolito L. Morandi A. Taddei M. L. Parri M. Comito G. Iscaro A. (2019). Cancer-associated fibroblasts promote prostate cancer malignancy via metabolic rewiring and mitochondrial transfer. *Oncogene* 38 5339–5355. 10.1038/s41388-019-0805-7 30936458

[B40] Jiang L. Shestov A. Swain P. Yang C. Parker S. Wang Q. (2016). Reductive carboxylation supports redox homeostasis during anchorage-independent growth. *Nature* 532 255–258. 10.1038/nature17393 27049945 PMC4860952

[B41] Jose C. Bellance N. Rossignol R. (2011). Choosing between glycolysis and oxidative phosphorylation: a tumor’s dilemma? *Biochim. Biophys. Acta* 1807 552–561. 10.1016/j.bbabio.2010.10.012 20955683

[B42] Juthberg S. K. Brismar T. (1997). Effect of metabolic inhibitors on membrane potential and ion conductance of rat astrocytes. *Cell Mol. Neurobiol.* 17 367–377. 10.1023/a:1026331226241 9262865 PMC11560238

[B43] Kawano S. Kojima M. Higuchi Y. Sugimoto M. Ikeda K. Sakuyama N. (2015). Assessment of elasticity of colorectal cancer tissue, clinical utility, pathological and phenotypical relevance. *Cancer Sci.* 106 1232–1239. 10.1111/cas.12720 26083008 PMC4582994

[B44] Kerr E. M. Gaude E. Turrell F. K. Frezza C. Martins C. P. (2016). Mutant Kras copy number defines metabolic reprogramming and therapeutic susceptibilities. *Nature* 531 110–113. 10.1038/nature16967 26909577 PMC4780242

[B45] Kim D. Wirtz D. (2011). Recapitulating cancer cell invasion in vitro. *Proc. Natl. Acad. Sci. U.S.A.* 108 6693–6694. 10.1073/pnas.1103983108 21490296 PMC3084149

[B46] LaGory E. L. Wu C. Taniguchi C. M. Ding C. C. Chi J. T. von Eyben R. (2015). Suppression of PGC-1alpha Is critical for reprogramming oxidative metabolism in renal cell carcinoma. *Cell Rep.* 12 116–127. 26119730 10.1016/j.celrep.2015.06.006 PMC4518559

[B47] Lark A. Livasy C. Dressler L. Moore D. Millikan R. Geradts J. (2005). High focal adhesion kinase expression in invasive breast carcinomas is associated with an aggressive phenotype. *Mod. Pathol.* 18 1289–1294. 10.1038/modpathol.3800424 15861214

[B48] LeBleu V. O’Connell J. Gonzalez Herrera K. Wikman H. Pantel K. Haigis M. (2014). PGC-1α mediates mitochondrial biogenesis and oxidative phosphorylation in cancer cells to promote metastasis. *Nat. Cell Biol.* 10 992–1003. 10.1038/ncb3039 25241037 PMC4369153

[B49] Lehuédé C. Dupuy F. Rabinovitch R. Jones R. Siegel P. (2016). Metabolic plasticity as a determinant of tumor growth and metastasis. *Cancer Res.* 76 5201–5208. 10.1158/0008-5472.CAN-16-0266 27587539

[B50] Li P. Liu Y. Liu W. Li G. Tang Q. Zhang Q. (2019). IR-783 inhibits breast cancer cell proliferation and migration by inducing mitochondrial fission. *Int. J. Oncol.* 55 415–424. 10.3892/ijo.2019.4821 31173174 PMC6615916

[B51] Li Y. Xu S. Li J. Zheng L. Feng M. Wang X. (2016). SIRT1 facilitates hepatocellular carcinoma metastasis by promoting PGC-1α-mediated mitochondrial biogenesis. *Oncotarget* 7 29255–29274. 10.18632/oncotarget.8711 27081083 PMC5045394

[B52] Lipinski K. A. Barber L. J. Davies M. N. Ashenden M. Sottoriva A. Gerlinger M. (2016). Cancer evolution and the limits of predictability in precision cancer medicine. *Trends Cancer* 2 49–63. 26949746 10.1016/j.trecan.2015.11.003 PMC4756277

[B53] Liu R. Zhang H. Zhang Y. Li S. Wang X. Wang X. (2017). Peroxisome proliferator-activated receptor gamma coactivator-1 alpha acts as a tumor suppressor in hepatocellular carcinoma. *Tumour Biol.* 39:1010428317695031. 10.1177/1010428317695031 28381162

[B54] Liu Y. Piao X. J. Xu W. T. Zhang Y. Zhang T. Xue H. (2021). Calycosin induces mitochondrial-dependent apoptosis and cell cycle arrest, and inhibits cell migration through a ROS-mediated signaling pathway in HepG2 hepatocellular carcinoma cells. *Toxicol. Vitro* 70:105052. 10.1016/j.tiv.2020.105052 33188878

[B55] Lu P. Weaver V. M. Werb Z. (2012). The extracellular matrix: a dynamic niche in cancer progression. *J. Cell Biol.* 196 395–406. 10.1083/jcb.201102147 22351925 PMC3283993

[B56] Luo C. Widlund H. Puigserver P. (2016b). PGC-1 coactivators: shepherding the mitochondrial biogenesis of tumors. *Trends Cancer* 2 619–631. 10.1016/j.trecan.2016.09.006 28607951 PMC5465638

[B57] Luo C. Lim J. H. Lee Y. Granter S. R. Thomas A. Vazquez F. (2016a). A PGC1alpha-mediated transcriptional axis suppresses melanoma metastasis. *Nature* 537 422–426. 10.1038/nature19347 27580028 PMC5161587

[B58] Luo Y. Zeng A. Fang A. Song L. Fan C. Zeng C. (2019). Nifuroxazide induces apoptosis, inhibits cell migration and invasion in osteosarcoma. *Invest. New Drugs* 37 1006–1013. 10.1007/s10637-019-00724-4 30680584

[B59] Ma H. Zhang J. Zhou L. Wen S. Tang H. Y. Jiang B. (2020). c-Src promotes tumorigenesis and tumor progression by activating PFKFB3. *Cell Rep.* 30 4235–4249. 10.1016/j.celrep.2020.03.005 32209481

[B60] Martinez J. Smith P. C. (2021). The dynamic interaction between extracellular matrix remodeling and breast tumor progression. *Cells* 10:1046. 10.3390/cells10051046 33946660 PMC8145942

[B61] Massagué J. Obenauf A. C. (2016). Metastatic colonization by circulating tumour cells. *Nature* 529 298–306. 10.1038/nature17038 26791720 PMC5029466

[B62] McGuirk S. Gravel S. Deblois G. Papadopoli D. Faubert B. Wegner A. (2013). PGC-1α supports glutamine metabolism in breast cancer. *Cancer Metab.* 1:22. 10.1186/2049-3002-1-22 24304688 PMC4178216

[B63] Metallo C. M. Gameiro P. A. Bell E. L. Mattaini K. R. Yang J. Hiller K. (2012). Reductive glutamine metabolism by IDH1 mediates lipogenesis under hypoxia. *Nature* 481 380–384. 10.1038/nature10602 22101433 PMC3710581

[B64] Mitra S. Schlaepfer D. (2006). Integrin-regulated FAK-Src signaling in normal and cancer cells. *Curr. Opin. Cell Biol.* 18 516–523. 16919435 10.1016/j.ceb.2006.08.011

[B65] Moldogazieva N. T. Mokhosoev I. M. Terentiev A. A. (2020). Metabolic heterogeneity of cancer cells: an interplay between HIF-1, GLUTs, and AMPK. *Cancers* 12:862. 10.3390/cancers12040862 32252351 PMC7226606

[B66] Mullen A. R. Wheaton W. W. Jin E. S. Chen P.-H. Sullivan L. B. Cheng T. (2012). Reductive carboxylation supports growth in tumour cells with defective mitochondria. *Nature* 481 385–388. 10.1038/nature10642 22101431 PMC3262117

[B67] Northcott J. M. Dean I. S. Mouw J. K. Weaver V. M. (2018). Feeling stress: the mechanics of cancer progression and aggression. *Front. Cell Dev. Biol.* 6:17. 10.3389/fcell.2018.00017 29541636 PMC5835517

[B68] Onodera Y. Nam J. M. Horikawa M. Shirato H. Sabe H. (2018). Arf6-driven cell invasion is intrinsically linked to TRAK1-mediated mitochondrial anterograde trafficking to avoid oxidative catastrophe. *Nat. Commun.* 9:2682. 10.1038/s41467-018-05087-7 29992963 PMC6041267

[B69] Pacheco-Velázquez S. C. Robledo-Cadena D. X. Hernández-Reséndiz I. Gallardo-Pérez J. C. Moreno-Sánchez R. Rodríguez-Enríquez S. (2018). Energy metabolism drugs block triple negative breast metastatic cancer cell phenotype. *Mol. Pharm.* 15 2151–2164. 29746779 10.1021/acs.molpharmaceut.8b00015

[B70] Padera T. P. Stoll B. R. Tooredman J. B. Capen D. di Tomaso E. Jain R. K. (2004). Pathology: cancer cells compress intratumour vessels. *Nature* 427:695. 10.1038/427695a 14973470

[B71] Papalazarou V. Zhang T. Paul N. R. Juin A. Cantini M. Maddocks O. D. K. (2020). The creatine-phosphagen system is mechanoresponsive in pancreatic adenocarcinoma and fuels invasion and metastasis. *Nat. Metab.* 2 62–80. 10.1038/s42255-019-0159-z 32694686 PMC7617069

[B72] Park K. S. Jo I. Pak K. Bae S. W. Rhim H. Suh S. H. (2002). FCCP depolarizes plasma membrane potential by activating proton and Na+ currents in bovine aortic endothelial cells. *Pflugers Arch.* 443 344–352. 10.1007/s004240100703 11810202

[B73] Parsons J. Horwitz A. Schwartz M. (2010). Cell adhesion: integrating cytoskeletal dynamics and cellular tension. *Nat. Rev. Mol. Cell Biol.* 11 633–643. 10.1038/nrm2957 20729930 PMC2992881

[B74] Pascual G. Avgustinova A. Mejetta S. Martín M. Castellanos A. Attolini C. (2017). Targeting metastasis-initiating cells through the fatty acid receptor CD36. *Nature* 541 41–45. 10.1038/nature20791 27974793

[B75] Phuyal S. Farhan H. (2019). Multifaceted Rho GTPase signaling at the endomembranes. *Front. Cell Dev. Biol.* 7:127. 10.3389/fcell.2019.00127 31380367 PMC6646525

[B76] Pickup M. W. Mouw J. K. Weaver V. M. (2014). The extracellular matrix modulates the hallmarks of cancer. *EMBO Rep.* 15 1243–1253. 10.15252/embr.201439246 25381661 PMC4264927

[B77] Piskounova E. Agathocleous M. Murphy M. M. Hu Z. Huddlestun S. E. Zhao Z. (2015). Oxidative stress inhibits distant metastasis by human melanoma cells. *Nature* 527 186–191. 10.1038/nature15726 26466563 PMC4644103

[B78] Ponce I. Garrido N. Tobar N. Melo F. Smith P. C. Martínez J. (2021). Matrix stiffness modulates metabolic interaction between human stromal and breast cancer cells to stimulate epithelial motility. *Metabolites* 11:432. 10.3390/metabo11070432 PMC8308000 34357326

[B79] Porporato P. Payen V. Pérez-Escuredo J. De Saedeleer C. Danhier P. Copetti T. (2014). A mitochondrial switch promotes tumor metastasis. *Cell Rep.* 8 754–766. 10.1016/j.celrep.2014.06.043 25066121

[B80] Porporato P. Sonveaux P. (2014). Paving the way for therapeutic prevention of tumor metastasis with agents targeting mitochondrial superoxide. *Mol. Cell Oncol.* 2:e968043. 10.4161/23723548.2014.968043 27308448 PMC4905283

[B81] Prasad P. Roy S. S. (2021). Glutamine regulates ovarian cancer cell migration and invasion through ETS1. *Heliyon* 7:e07064. 10.1016/j.heliyon.2021.e07064 34136678 PMC8180613

[B82] Primeau A. J. Rendon A. Hedley D. Lilge L. Tannock I. F. (2005). The distribution of the anticancer drug Doxorubicin in relation to blood vessels in solid tumors. *Clin. Cancer Res.* 11(24 Pt 1) 8782–8788. 10.1158/1078-0432.Ccr-05-1664 16361566

[B83] Provenzano P. P. Eliceiri K. W. Campbell J. M. Inman D. R. White J. G. Keely P. J. (2006). Collagen reorganization at the tumor-stromal interface facilitates local invasion. *BMC Med.* 4:38. 10.1186/1741-7015-4-38 17190588 PMC1781458

[B84] Provenzano P. P. Inman D. R. Eliceiri K. W. Knittel J. G. Yan L. Rueden C. T. (2008). Collagen density promotes mammary tumor initiation and progression. *BMC Med.* 6:11. 10.1186/1741-7015-6-11 18442412 PMC2386807

[B85] Ravi S. Chacko B. Sawada H. Kramer P. A. Johnson M. S. Benavides G. A. (2015). Metabolic plasticity in resting and thrombin activated platelets. *PLoS One* 10:e0123597. 10.1371/journal.pone.0123597 25875958 PMC4395425

[B86] Riggi N. Aguet M. Stamenkovic I. (2018). Cancer metastasis: a reappraisal of its underlying mechanisms and their relevance to treatment. *Annu. Rev. Pathol.* 13 117–140. 10.1146/annurev-pathol-020117-044127 29068753

[B87] Rinaldi G. Rossi M. Fendt S. M. (2018). Metabolic interactions in cancer: cellular metabolism at the interface between the microenvironment, the cancer cell phenotype and the epigenetic landscape. *Wiley Interdiscip. Rev. Syst. Biol. Med.* 10:e1397. 10.1002/wsbm.1397 28857478

[B88] Ryu D. Lee J. H. Kwak M. K. (2020). NRF2 level is negatively correlated with TGF-β1-induced lung cancer motility and migration via NOX4-ROS signaling. *Arch. Pharm. Res.* 43 1297–1310. 10.1007/s12272-020-01298-z 33242180

[B89] Sancho P. Burgos-Ramos E. Tavera A. Bou Kheir T. Jagust P. Schoenhals M. (2015). MYC/PGC-1α balance determines the metabolic phenotype and plasticity of pancreatic cancer stem cells. *Cell Metab.* 22 590–605. 10.1016/j.cmet.2015.08.015 26365176

[B90] Scheid A. D. Beadnell T. C. Welch D. R. (2021). Roles of mitochondria in the hallmarks of metastasis. *Br. J. Cancer* 124 124–135. 10.1038/s41416-020-01125-8 33144695 PMC7782743

[B91] Schild T. Low V. Blenis J. Gomes A. (2018). Unique metabolic adaptations dictate distal organ-specific metastatic colonization. *Cancer Cell* 33 347–354. 10.1016/j.ccell.2018.02.001 29533780 PMC5889305

[B92] Schuler M. H. Lewandowska A. Caprio G. D. Skillern W. Upadhyayula S. Kirchhausen T. (2017). Miro1-mediated mitochondrial positioning shapes intracellular energy gradients required for cell migration. *Mol. Biol. Cell* 28 2159–2169. 10.1091/mbc.E16-10-0741 28615318 PMC5531732

[B93] Si L. Fu J. Liu W. Hayashi T. Nie Y. Mizuno K. (2020). Silibinin inhibits migration and invasion of breast cancer MDA-MB-231 cells through induction of mitochondrial fusion. *Mol. Cell Biochem.* 463 189–201. 10.1007/s11010-019-03640-6 31612353

[B94] Simpson C. Anyiwe K. Schimmer A. (2008). Anoikis resistance and tumor metastasis. *Cancer Lett.* 272 177–185. 10.1016/j.canlet.2008.05.029 18579285

[B95] Song X. Wang Z. Liang H. Zhang W. Ye Y. Li H. (2017). Dioscin induces gallbladder cancer apoptosis by inhibiting ROS-mediated PI3K/AKT signalling. *Int. J. Biol. Sci.* 13 782–793. 10.7150/ijbs.18732 28656003 PMC5485633

[B96] Tennakoon J. B. Shi Y. Han J. J. Tsouko E. White M. A. Burns A. R. (2014). Androgens regulate prostate cancer cell growth via an AMPK-PGC-1alpha-mediated metabolic switch. *Oncogene* 33 5251–5261. 24186207 10.1038/onc.2013.463 PMC4009392

[B97] Tian C. Clauser K. R. Öhlund D. Rickelt S. Huang Y. Gupta M. (2019). Proteomic analyses of ECM during pancreatic ductal adenocarcinoma progression reveal different contributions by tumor and stromal cells. *Proc. Natl. Acad. Sci. U.S.A.* 116 19609–19618. 10.1073/pnas.1908626116 31484774 PMC6765243

[B98] Torrano V. Valcarcel-Jimenez L. Cortazar A. R. Liu X. Urosevic J. Castillo-Martin M. (2016). The metabolic co-regulator PGC1alpha suppresses prostate cancer metastasis. *Nat. Cell Biol.* 18 645–656. 10.1038/ncb3357 27214280 PMC4884178

[B99] Urra F. Muñoz F. Córdova-Delgado M. Ramírez M. Peña-Ahumada B. Rios M. (2018). FR58P1a; a new uncoupler of OXPHOS that inhibits migration in triple-negative breast cancer cells via Sirt1/AMPK/β1-integrin pathway. *Sci. Rep.* 8:13190. 10.1038/s41598-018-31367-9 30181620 PMC6123471

[B100] Urra F. Muñoz F. Lovy A. Cárdenas C. (2017). The mitochondrial complex(I)ty of cancer. *Front. Oncol.* 7:118. 10.3389/fonc.2017.00118 28642839 PMC5462917

[B101] Urra F. A. Weiss-López B. Araya-Maturana R. (2016b). Determinants of anti-cancer effect of mitochondrial electron transport chain inhibitors: bioenergetic profile and metabolic flexibility of cancer cells. *Curr. Pharm. Des.* 22 5998–6008. 10.2174/1381612822666160719122626 27510477

[B102] Urra F. A. Cordova-Delgado M. Lapier M. Orellana-Manzano A. Acevedo-Arevalo L. Pessoa-Mahana H. (2016a). Small structural changes on a hydroquinone scaffold determine the complex I inhibition or uncoupling of tumoral oxidative phosphorylation. *Toxicol. Appl. Pharmacol.* 291 46–57. 10.1016/j.taap.2015.12.005 26712467

[B103] Urra F. A. Araya-Maturana R. (2017). Targeting metastasis with snake toxins: molecular mechanisms. *Toxins* 9:390. 10.3390/toxins9120390 29189742 PMC5744110

[B104] Urra F. A. Araya-Maturana R. (2020). Putting the brakes on tumorigenesis with snake venom toxins: new molecular insights for cancer drug discovery. *Semin. Cancer Biol.* 15:30. 10.1016/j.semcancer.2020.05.006 32428714

[B105] Urra F. A. Fuentes-Retamal S. Palominos C. Araya-Maturana R. (2021). “Recent advances in molecular mechanisms of anticancer natural products that target mitochondrial bioenergetics,” in *Studies in Natural Products Chemistry*, ed. Atta-ur-Rahman A. (Amsterdam: Elsevier), 1–41.

[B106] Urra F. A. Martinez-Cifuentes M. Pavani M. Lapier M. Jana-Prado F. Parra E. (2013). An ortho-carbonyl substituted hydroquinone derivative is an anticancer agent that acts by inhibiting mitochondrial bioenergetics and by inducing G(2)/M-phase arrest in mammary adenocarcinoma TA3. *Toxicol. Appl. Pharmacol.* 267 218–227. 10.1016/j.taap.2012.12.023 23333614

[B107] Valcarcel-Jimenez L. Gaude E. Torrano V. Frezza C. Carracedo A. (2017). Mitochondrial metabolism: yin and yang for tumor progression. *Trends Endocrinol. Metab.* 28 748–757. 10.1016/j.tem.2017.06.004 28938972 PMC6047739

[B108] Vatrinet R. Leone G. De Luise M. Girolimetti G. Vidone M. Gasparre G. (2017). The α-ketoglutarate dehydrogenase complex in cancer metabolic plasticity. *Cancer Metab.* 5:3. 10.1186/s40170-017-0165-0 28184304 PMC5289018

[B109] Vazquez F. Lim J. H. Chim H. Bhalla K. Girnun G. Pierce K. (2013). PGC1alpha expression defines a subset of human melanoma tumors with increased mitochondrial capacity and resistance to oxidative stress. *Cancer Cell* 23 287–301. 10.1016/j.ccr.2012.11.020 23416000 PMC3708305

[B110] Visavadiya N. P. Keasey M. P. Razskazovskiy V. Banerjee K. Jia C. Lovins C. (2016). Integrin-FAK signaling rapidly and potently promotes mitochondrial function through STAT3. *Cell Commun. Signal.* 14:32. 10.1186/s12964-016-0157-7 27978828 PMC5159999

[B111] Vivas-Ruiz D. E. Sandoval G. A. Gonzalez-Kozlova E. Zarria-Romero J. Lazo F. Rodriguez E. (2020). Fibrinogen-clotting enzyme, pictobin, from Bothrops pictus snake venom. Structural and functional characterization. *Int. J. Biol. Macromol.* 153 779–795. 10.1016/j.ijbiomac.2020.03.055 32169454

[B112] Winkler J. Abisoye-Ogunniyan A. Metcalf K. J. Werb Z. (2020). Concepts of extracellular matrix remodelling in tumour progression and metastasis. *Nat. Commun.* 11:5120. 10.1038/s41467-020-18794-x 33037194 PMC7547708

[B113] Wu Y. Zanotelli M. R. Zhang J. Reinhart-King C. A. (2021). Matrix-driven changes in metabolism support cytoskeletal activity to promote cell migration. *Biophys. J.* 120 1705–1717. 10.1016/j.bpj.2021.02.044 33705759 PMC8204337

[B114] Wynn M. L. Yates J. A. Evans C. R. Van Wassenhove L. D. Wu Z. F. Bridges S. (2016). RhoC GTPase is a potent regulator of glutamine metabolism and N-Acetylaspartate production in inflammatory breast cancer cells. *J. Biol. Chem.* 291 13715–13729. 10.1074/jbc.M115.703959 27129239 PMC4919454

[B115] Yan H. Ren M. Y. Wang Z. X. Feng S. J. Li S. Cheng Y. (2017). Zerumbone inhibits melanoma cell proliferation and migration by altering mitochondrial functions. *Oncol. Lett.* 13 2397–2402. 10.3892/ol.2017.5742 28454410 PMC5403190

[B116] Yang L. Moss T. Mangala L. Marini J. Zhao H. Wahlig S. (2014). Metabolic shifts toward glutamine regulate tumor growth, invasion and bioenergetics in ovarian cancer. *Mol. Syst. Biol.* 10:728. 10.1002/msb.20134892 24799285 PMC4188042

[B117] Yang S. Liao Y. Li L. Xu X. Cao L. (2018). Zeylenone induces mitochondrial apoptosis and inhibits migration and invasion in gastric cancer. *Molecules* 23:2149. 10.3390/molecules23092149 30150551 PMC6225419

[B118] Yang Y. Wang Y. Che X. Hou K. Wu J. Zheng C. (2021). Integrin α5 promotes migration and invasion through the FAK/STAT3/AKT signaling pathway in icotinib-resistant non-small cell lung cancer cells. *Oncol. Lett.* 22:556. 10.3892/ol.2021.12817 34084223 PMC8161469

[B119] Yun C. W. Lee J. H. Lee S. H. (2019). Hypoxia-induced PGC-1alpha regulates mitochondrial function and tumorigenesis of colorectal cancer cells. *Anticancer Res.* 39 4865–4876. 10.21873/anticanres.13672 31519589

[B120] Zanotelli M. R. Zhang J. Reinhart-King C. A. (2021). Mechanoresponsive metabolism in cancer cell migration and metastasis. *Cell Metab.* 33 1307–1321. 10.1016/j.cmet.2021.04.002 33915111 PMC9015673

